# Cannabinoids As Potential Treatment for Chemotherapy-Induced Nausea and Vomiting

**DOI:** 10.3389/fphar.2016.00221

**Published:** 2016-07-26

**Authors:** Erin M. Rock, Linda A. Parker

**Affiliations:** Department of Psychology and Collaborative Neuroscience Graduate Program, University of GuelphGuelph, ON, Canada

**Keywords:** cannabinoid, acute nausea, anticipatory nausea, vomiting, conditioned gaping

## Abstract

Despite the advent of classic anti-emetics, chemotherapy-induced nausea is still problematic, with vomiting being somewhat better managed in the clinic. If post-treatment nausea and vomiting are not properly controlled, anticipatory nausea—a conditioned response to the contextual cues associated with illness-inducing chemotherapy—can develop. Once it develops, anticipatory nausea is refractive to current anti-emetics, highlighting the need for alternative treatment options. One of the first documented medicinal uses of Δ^9^-tetrahydrocannabinol (Δ^9^-THC) was for the treatment of chemotherapy-induced nausea and vomiting (CINV), and recent evidence is accumulating to suggest a role for the endocannabinoid system in modulating CINV. Here, we review studies assessing the therapeutic potential of cannabinoids and manipulations of the endocannabinoid system in human patients and pre-clinical animal models of nausea and vomiting.

## Introduction

*Cannabis sativa* has been used as a medicine for centuries (see Hanus and Mechoulam, 2005; Iversen, 2008). It was not until the 1970's that oncologists demonstrated that smoked cannabis attenuated chemotherapy-induced nausea and vomiting (CINV). Few clinical trials have compared the efficacy of cannabis-based medicines with the currently recommended anti-emetic regimen, or as an adjunct to this treatment. We review findings on the potential of exogenous cannabinoids and manipulations of the endogenous cannabinoid system to reduce acute and anticipatory CINV.

## Chemotherapy-induced nausea and vomiting (CINV)

Chemotherapy patients experience acute nausea and vomiting (occurring up to 24 h post-treatment; Fiore and Gralla, 1984). If improperly managed, this post-treatment CINV can lead to anticipatory nausea and vomiting; a conditioned nausea response upon re-exposure to the chemotherapy clinic (Morrow, 1982). Current guidelines to manage highly emetogenic acute CINV recommend a three-drug regimen of the 5-hydroxytryptamine 3 (5-HT_3_) receptor antagonist (such as ondansetron), along with dexamethasone, and a neurokinin 1 (NK_1_) receptor antagonist (such as aprepitant) *before* beginning chemotherapy (Roila et al., 2010). Even with this standard treatment acute nausea is still problematic (no acute nausea reported in 66% of patients; Kim et al., 2015). None of these treatments are effective in reducing anticipatory nausea (e.g., Roscoe et al., 2000), with sedating benzodiazepines currently prescribed (Razavi et al., 1993; Malik et al., 1995). Therefore, nausea (acute and anticipatory) continues to be problematic.

## Cannabinoids in human CINV

Because current treatments cannot properly manage CINV, alternatives including constituents of the cannabis plant and modulation of the endogenous cannabinoid system, have been investigated.

### Effect of Δ^9^-THC and Δ^9^-THC-like synthetics

One of the few recognized medicinal effects of the cannabis plant is the control of CINV, by Δ^9^-THC, the psychoactive compound in cannabis (Gaoni and Mechoulam, 1964). Synthetic Δ^9^-THC is available for treatment of CINV in capsule form as dronabinol (Marinol®), or nabilone (Cesamet®). Each of these compounds acts as a partial agonist of the cannabinoid 1 (CB_1_) and cannabinoid 2 (CB_2_) receptors. In comparison to placebo or the dopamine 2 (D_2_) receptor antagonists (anti-emetics which predated the 5-HT_3_ receptor antagonists), Δ^9^-THC or Δ^9^-THC-like synthetics are more effective in reducing acute CINV (Sallan et al., 1975; Chang et al., 1979; Ekert et al., 1979; Frytak et al., 1979; Herman et al., 1979; Kluin-Neleman et al., 1979; Orr et al., 1980; Steele et al., 1980; Einhorn et al., 1981; Orr and McKernan, 1981; Johansson et al., 1982; Jones et al., 1982; Levitt, 1982; Wada et al., 1982; Ahmedzai et al., 1983; Niamatali et al., 1984; Niiranen and Mattson, 1985; Dalzell et al., 1986; Niederle et al., 1986; Pomeroy et al., 1986; Chan et al., 1987; McCabe et al., 1988; Lane et al., 1990).

The only published clinical trial assessing the effect of dronabinol on anticipatory nausea showed that dronabinol was ineffective, although most patients were receiving highly emetogenic chemotherapy regimens (Lane et al., 1991). Therefore, dronabinol may be effective in reducing anticipatory nausea developing from less emetogenic chemotherapy regimens.

## Pre-clinical animal models of vomiting

Since rats and mice cannot vomit, species capable of vomiting are used in emesis research. *Suncus murinus* (house musk shrew) or *Cryptotis parva* (least shrew) vomit to toxins such as nicotine (Matsuki et al., 1988, 1990; Torii et al., 1991; Nakayama et al., 2005; Parker et al., 2009; Rock et al., 2012), the chemotherapeutic agent cisplatin (Matsuki et al., 1988, 1990; Torii et al., 1991; Darmani, 1998, 2001b; Sam et al., 2003; Lau et al., 2005; Parker et al., 2009; Ray et al., 2009; Rock et al., 2012), or lithium chloride (LiCl; e.g., Parker et al., 2004). Ferrets also vomit following cisplatin or morphine 6 glucuronide (M6G; Van Sickle et al., 2001, 2003; Sharkey et al., 2007). These species have therefore been used to study emesis. Please refer to Table 1 for details regarding the findings of exogenous cannabinoids and manipulations of the endogenous cannabinoid system on vomiting in animal models.

**Table 1:** **Effect of exogenous cannabinoids and manipulations of the endogenous cannabinoid system on vomiting in animal models**.

**Compound**	**Species**	**Dose**	**Emetogenic agent**	**Effect on emesis**	**Receptor mediation**	**References**
Δ^9^**-THC, THCA, AND** Δ^9^**-THC-LIKE SYNTHETICS**						
Δ^9^-THC	Least shrew	20 mg/kg, i.p.	SR141716	Reduced	CB_1_	Darmani, 2001a
		0.25, 0.5, 1, 2.5, 5, 10 mg/kg, i.p.	Cisplatin	Reduced		Darmani, 2001b; Ray et al., 2009; Wang et al., 2009
		5, 10 mg/kg, i.p.	D_2_/D_3_ receptor agonists	Reduced		Darmani and Crim, 2005
Δ^9^-THC + tropisetron	Least shrew	0.25, 0.5 mg/kg, i.p.	Cisplatin	Enhanced reduction	Not evaluated	Wang et al., 2009
CP 55, 940	Least shrew	1 mg/kg, i.p.	SR141716	Reduced	CB_1_	Darmani, 2001a
WIN 55, 212-2	Least shrew	10 mg/kg, i.p.	SR141716	Reduced	CB_1_	Darmani, 2001a
Δ^9^-THC	House Musk Shrew	3–20 mg/kg, i.p.	LiCl	Reduced	CB_1_	Parker et al., 2004
		2.5, 5, 10 mg/kg, i.p.	Cisplatin	Reduced	Not evaluated	Kwiatkowska et al., 2004
Δ^9^-THC + ondansetron	House Musk Shrew	1.25 mg/kg, i.p.	Cisplatin	Enhanced reduction	Not evaluated	Kwiatkowska et al., 2004
Δ^9^-THC	Ferret	0.5, 1 mg/kg, i.p.	Cisplatin	Reduced	CB_1_	Van Sickle et al., 2003
		1 mg/kg, i.p.	M6G	Reduced		Van Sickle et al., 2001
THCA	House musk shrew	0.05, 0.5 mg/kg, i.p	LiCl	Reduced	CB_1_	Rock et al., 2013
Nabilone	Dog	0.1 mg/kg, i.v.	Cisplatin	No effect	Not evaluated	Gylys et al., 1979
	Cat	0.1 mg/kg, i.v.	Apomorphine, deslanoside	Reduced	Not evaluated	London et al., 1979
		2.7 × 10^−7^ mole/kg, i.v.	Cisplatin			McCarthy and Borison, 1981
**CBD AND CBDA**						
CBD	House musk shrew	5, 10 mg/kg, i.p. or 5, 10 mg/kg, s.c.	LiCl, nicotine, cisplatin	Reduced	5-HT_1A_	Kwiatkowska et al., 2004; Parker et al., 2004; Rock et al., 2012
CBD	House musk shrew	25, 40 mg/kg, i.p.	LiCl, cisplatin	Increased	Not evaluated	Kwiatkowska et al., 2004; Parker et al., 2004
CBD + THC	House musk shrew	CBD (2.5 mg/kg, i.p.), THC (1 mg/kg, i.p.)	LiCl	Enhanced reduction	Not evaluated	Rock and Parker, 2015
CBDA	House musk shrew	0.1, 0.5 mg/kg, i.p.	LiCl, cisplatin	Reduced	Not evaluated	Bolognini et al., 2013
CBDA + THC	House musk shrew	CBDA (0.05 mg/kg, i.p.), THC (1 mg/kg, i.p.)	LiCl	Enhanced reduction	Not evaluated	Rock and Parker, 2015
**AEA AND FAAH INHIBITION**						
AEA	Ferret	1, 2 mg/kg, i.p.	M6G	Reduced	CB_1_ TRPV1	Van Sickle et al., 2005; Sharkey et al., 2007
URB597	Ferret	3, 5 mg/kg, i.p.	M6G	Reduced	TRPV1 or CB_1_	Van Sickle et al., 2005; Sharkey et al., 2007
URB597	House Musk Shrew	0.9 mg/kg, i.p.	Cisplatin, nicotine	Reduced	CB_1_	Parker et al., 2009
AA-5-HT	Least shrew	10 mg/kg, i.p.	Itself	Produced	Not evaluated	Darmani et al., 2005
URB597		20 mg/kg, i.p.				
AA-5-HT		2.5, 5 mg/kg	Cisplatin	No effect	Not evaluated	Darmani et al., 2005
URB597		5, 10 mg/kg, i.p.				
**2-AG AND MAGL INHIBITION**						
2-AG	Least shrew	2.5, 5, 10 mg/kg, i.p.	Itself	Produced	CB_1_	Darmani, 2001c
2-AG	House musk shrew	2, 5 mg/kg, i.p.	LiCl	Reduced	Non-CB_1_	Sticht et al., 2013
JZL184	House musk shrew	16, 40 mg/kg, i.p.	LiCl	Reduced	CB_1_	Sticht et al., 2013
MJN110		10, 20 mg/kg, i.p.			CB_1_	Parker et al., 2015
2-AG	Ferret	1, 2 mg/kg, i.p.	M6G	Reduced	CB_1_ CB_2_ TRPV1	Van Sickle et al., 2005; Sharkey et al., 2007

### Effect of Δ^9^-THC, tetrahydrocannabinolic acid (THCA), and Δ^9^-THC-like synthetics on vomiting

In the least shrew, CB_1_ receptor agonists such as Δ^9^-THC (20 mg/kg, i.p.) reduced vomiting induced by the CB_1_ receptor antagonist/inverse agonist, SR141716 (20 mg/kg, intraperitoneal, i.p.; Darmani, 2001a). As well, Δ^9^-THC (20 mg/kg, i.p.) reduced cisplatin-induced vomiting, and this effect was reversed by SR141716 [10 mg/kg, subcutaneous (s.c.) or 2 mg/kg, i.p.] in the least shrew (Darmani, 2001b; Ray et al., 2009; Wang et al., 2009). In the house musk shrew, Δ^9^-THC (2.5–20 mg/kg, i.p.) also reduced LiCl- and cisplatin-induced vomiting, these effects were blocked by SR141716 (2.5 mg/kg, i.p.) (Kwiatkowska et al., 2004; Parker et al., 2004). In ferrets, Δ^9^-THC (0.5, 1 mg/kg, i.p.) reduced cisplatin-, or M6G-induced vomiting, these effects were blocked by SR141716 (5 mg/kg, i.p.; Van Sickle et al., 2003) or AM251 (5 mg/kg, i.p.; Van Sickle et al., 2001). In addition, Δ^9^-THC's precursor tetrahydrocannabinolic acid (THCA), present in fresh cannabis and decarboxylated upon heating or drying of the plant, (0.05, 0.5 mg/kg, i.p.) reduced LiCl-induced vomiting, an effect reversed by SR141716 (2.5 mg/kg, i.p.; Rock et al., 2013). These results complement human findings that Δ^9^-THC is anti-emetic, exerting its effect via the CB_1_ receptor.

### Effect of cannabidiol (CBD) and cannabidiolic acid (CBDA) on vomiting

For another non-psychoactive cannabinoid, cannabidiol (CBD) low doses (5, 10 mg/kg, i.p) reduced, but high doses (20–40 mg/kg, i.p.) potentiated LiCl-, nicotine-, and cisplatin-induced vomiting in house musk shrews (Kwiatkowska et al., 2004; Parker et al., 2004). Suppression of vomiting by CBD at low doses (5, 10 mg/kg, s.c.) was blocked by a 5-hydroxytryptamine 1A (5-HT_1A_) receptor antagonist (Rock et al., 2012). CBD's precursor cannabidiolic acid (CBDA), is decarboxylated when the fresh cannabis plant is heated or dried. In house musk shrews, CBDA (0.1, 0.5 mg/kg, i.p.) reduced LiCl-, and cisplatin-induced emesis (Bolognini et al., 2013). These findings suggest that CBD and CBDA are anti-emetic in a dose-dependent manner, with CBDA being more potent.

### Effect of anandamide (AEA) and FAAH inhibition on vomiting

The endogenous cannabinoid, anandamide (AEA), produced and released on-demand, is rapidly degraded by fatty acid amide hydrolase (FAAH). As well, FAAH degrades other fatty acids including oleoylethanolamide (OEA) and palmitoylethanolamine (PEA), which act on peroxisome proliferator-activated receptor alpha (PPARα), instead of CB_1_ or CB_2_ receptors. Interestingly, Venkatesan et al. (2016) reported increased levels of serum OEA and PEA (with a trend toward increased AEA and 2-AG) while patients were experiencing cyclic vomiting. On the other hand, no differences in plasma AEA, OEA or PEA were detected in pregnant women experiencing hyperemesis gravidarum—severe nausea and vomiting (Gebeh et al., 2014).

In animal models, AEA (1, 2 mg/kg, i.p.) reduced M6G-induced emesis in ferrets, an effect blocked by a transient receptor potential cation channel subfamily V member 1 (TRPV1) receptor antagonist (Sharkey et al., 2007) or AM251 (5 mg/kg, i.p.; Van Sickle et al., 2005). The FAAH inhibitor, URB597 (3, 5 mg/kg, i.p.) also reduced M6G-induced emesis in ferrets, an effect blocked by AM251 (5 mg/kg, i.p.) or a TRPV1 receptor antagonist (Van Sickle et al., 2005; Sharkey et al., 2007) but a PPARα antagonist was not evaluated. URB597 (0.9 mg/kg, i.p.) also reduced nicotine-induced vomiting in house musk shrews, an effect blocked by SR141716 (2.5 mg/kg, i.p.; Parker et al., 2009). These results suggest the anti-emetic effects of AEA and FAAH inhibition are mediated by activation of the CB_1_ receptor. In ferrets, the TRPV1 receptor also plays a role, an effect not yet been evaluated in house musk shrews.

In comparison, administration of the FAAH inhibitors AA-5-HT (10 mg/kg, i.p.) or URB597 (20 mg/kg, i.p.) themselves induced emesis (Darmani et al., 2005); however 20 mg/kg of URB597 is a much higher dose than is typically given. These species-dependent effects of AEA in the modulation of emesis are puzzling, warranting further investigation.

### Effect of 2-AG and MAGL inhibition on vomiting

The endogenous cannabinoid 2-Arachidonoylglycerol (2-AG), produced and released on-demand, is rapidly degraded by monoacylglycerol lipase (MAGL). In least shrews, 2-AG (2.5, 5, 10 mg/kg, i.p.) produced emesis (Darmani, 2001c). Indeed, in response to cisplatin in least shrews, brain 2-AG levels increased, while gut 2-AG levels decreased (Darmani et al., 2005). This is interesting, as Choukèr et al. (2010) reported lower blood endocannabinoid levels among those experiencing motion sickness, and higher blood endocannabinoid levels among those not.

In contrast, in house musk shrews, 2-AG (1–10 mg/kg, i.p.) did not induce emesis. Instead, 2-AG (2, 5 mg/kg, i.p.) reduced LiCl-induced vomiting (Sticht et al., 2013). Furthermore, 2-AG (1, 2 mg/kg, i.p.) reduced M6G-induced emesis in ferrets, effects blocked by a TRPV1 receptor antagonist (Sharkey et al., 2007) or AM251 (5 mg/kg, i.p.; Van Sickle et al., 2005) or the CB_2_ receptor antagonist AM630 (5 mg/kg, i.p.; Van Sickle et al., 2005). Although AM630 did not block vomiting produced by M6G in ferrets, Rock et al. (2016) found that the CB_2_ receptor agonist, HU308 (2.5 and 5 mg/kg, i.p.) reduced LiCl-induced vomiting in house musk shrews, an effect that was reversed by the CB_2_ receptor antagonist, AM630 (3 mg/kg, i.p.). These results together suggest that CB_1_, CB_2_ and TRPV1 receptors play a role in the emetic response depending on species and emetic agent employed.

The selective MAGL inhibitor MJN110 (10, 20 mg/kg, i.p.) suppressed LiCl-induced vomiting in house musk shrews; an effect reversed by SR141716 (2.5 mg/kg, i.p.; Parker et al., 2015). These results suggest CB_1_ receptor activation for 2-AG's anti-emetic effect, but also suggest TRPV1 or CB_2_ receptor mediation in ferrets, effects not yet investigated in house musk shrews. Overall, these species-dependent effects involving 2-AG and AEA warrant further investigation.

## Conditioned gaping re-clinical models of nausea in rats

Use of pre-clinical animal models has led to a good understanding of emesis neurobiology (Hornby, 2001), but the brain circuits mediating nausea are still not well characterized (Andrews and Horn, 2006). Such nausea circuitry may be more complex than that of emesis (see Kenward et al., 2015). Emesis is a gastrointestinal event controlled by structures within the brainstem (Hornby, 2001), whereas nausea is thought to require forebrain activation (Sanger and Andrews, 2006; Horn, 2008; Holmes et al., 2009). Although the visceral inputs from the gastrointestinal tract to the brain have been identified (Cechetto and Saper, 1987), it is unclear how these inputs are processed in the forebrain to produce nausea, largely due to the lack of reliable animal models of nausea. Here we describe current animal models of nausea. For a complete review of these models please refer to Sharkey et al. (2014).

To evaluate potential anti-nausea compounds, selective pre-clinical animal models are necessary. One such model is conditioned gaping in rats. Please refer to Table 2 for details regarding the effects of exogenous cannabinoids and manipulations of the endogenous cannabinoid system in rat models of conditioned gaping.

**Table 2:** **Effect of exogenous cannabinoids and manipulations of the endogenous cannabinoid system on models of acute and anticipatory nausea in rats**.

**Compound**	**Dose details**	**Efficacy in acute nausea-induced gaping and receptor mediation**	**Efficacy in contextually elicited gaping and receptor mediation**
Δ^9^**-THC, THCA, AND** Δ^9^**-THC-LIKE SYNTHETICS**			
Δ^9^-THC	0.5, 1, 10 mg/kg, i.p. for acute; 0.5 mg/kg, i.p. for anticipatory	Reduced (Parker and Mechoulam, 2003; Rock et al., 2015a)	Reduced (Limebeer et al., 2006; Rock et al., 2014)
HU210	0.001, 0.005 mg/kg, i.p.	Reduced, CB_1_(Parker and Mechoulam, 2003)	Not evaluated
THCA	0.05, 0.5 mg/kg, i.p. for acute; 0.05 mg/kg, i.p. for anticipatory	Reduced (Rock et al., 2013)	Reduced, CB_1_(Rock et al., 2013)
**CBD AND CBDA**			
CBD	5 mg/kg, i.p. or s.c. for acute; 1, 5 mg/kg, i.p. for anticipatory	Reduced, 5-HT_1A_ (Parker and Mechoulam, 2003; Rock et al., 2012)	Reduced (Rock et al., 2008)
CBDA	0.5 μg/kg–0.1 mg/kg, i.p. for acute; 0.001, 0.01, 0.1 mg/kg, i.p. for anticipatory	Reduced, 5-HT_1A_ (Bolognini et al., 2013; Rock and Parker, 2013a; Rock et al., 2015a)	Reduced, 5-HT_1A_ (Bolognini et al., 2013; Rock et al., 2014)
CBDA + Δ^9^-THC	CBDA (0.01 and 0.1 μg/kg) + Δ^9^-THC (0.01 and 0.1 mg/kg) for acute; CBDA (1.0, 10 μg/kg, i.p.) + Δ^9^-THC (1.0, 10 mg/kg, i.p.) for anticipatory	Enhanced Reduction (Rock et al., 2015a)	Reduced (Rock et al., 2015a)
CBDA + THCA	CBDA (0.1 μg/kg, i.p.) + THCA (5 μg/kg, i.p.)	Not evaluated	Enhanced reduction, 5-HT_1A_ or CB_1_(Rock et al., 2014)
CBDA + ondansetron	CBDA (0.1 μg/kg, i.p.) + ondansetron (1 μg/kg, i.p.)	Enhanced Reduction (Rock and Parker, 2013a)	Not evaluated
CBDA + D_2_ receptor antagonist	CBDA (0.1 μg/kg, i.p.) + D_2_ antagonist (0.3 mg/kg, s.c.)	Enhanced Reduction (Rock and Parker, 2013b)	Not evaluated
**AEA AND FAAH INHIBITION**			
AEA	5 mg/kg, i.p.	No effect (Cross-Mellor et al., 2007)	Not evaluated
	0.4 μg into the IC	No effect (Sticht et al., 2015)	Not evaluated
AEA + URB597	AEA (0.4 μg) + URB597 (0.01 μg) into the IC	Reduced (Sticht et al., 2015)	Not evaluated
URB597	0.3, 10 mg/kg, i.p.	No effect (Rock et al., 2015b)	Reduced, CB_1_(Rock et al., 2008)
	(0.01 μg) into the IC	No effect (Sticht et al., 2016)	Not evaluated
PF3845	10 mg/kg, i.p. for acute; 10, 20 mg/kg, i.p. for anticipatory	Reduced, PPARα (Rock et al., 2015b)	Reduced, CB_1_(Rock et al., 2015b)
	2 μg into the IC	No effect (Sticht et al., 2016)	No effect (Limebeer et al., 2016)
AM4303	20 mg/kg, i.p.	Reduced (Parker et al., 2016)	Reduced (Parker et al., 2016)
**2-AG AND MAGL INHIBITION**			
2-AG	1.25, 2 mg/kg, i.p. for acute	Reduced, COX (Sticht et al., 2012)	Not evaluated
2-AG + JZL184	JZL184 (40 mg/kg, i.p.) + 2-AG (2 mg/kg, i.p.)	Reduced, CB_1_(Sticht et al., 2012)	Not evaluated
MJN110	10, 20 mg/kg, i.p.	Reduced, CB_1_(Parker et al., 2015)	Reduced, CB_1_(Parker et al., 2015)
	2 μg into the IC	Reduced, CB_1_(Sticht et al., 2016)	Reduced, CB_1_(Limebeer et al., 2016)
AM4301	20 mg/kg, i.p.	Reduced, CB_1_(Parker et al., 2016)	Reduced (Parker et al., 2016)
	2 μg into the IC	Reduced (Parker et al., 2016)	Not evaluated
**DUAL FAAH/MAGL INHIBITION**			
JZL195	10 mg/kg, i.p. for anticipatory	Not evaluated	Reduced, CB_1_(Limebeer et al., 2014)
	10 μg into the IC	Reduced (Sticht et al., 2016)	Not evaluated
AM4302	20 mg/kg, i.p. for acute; 5, 10, 20 mg/kg, i.p. for anticipatory	Reduced (Parker et al., 2016)	Reduced, CB_1_ (Parker et al., 2016)

*Δ^9^-THC, Δ^9^-tetrahydrocannabinol; 5-HT_3_, 2-AG, 2-Arachidonoylglycerol; 5-hydroxytryptamine 3; AEA, anandamide; CB_1,_cannabinoid 1; CB_2_, cannabinoid 2; CBD, cannabidiol; CBDA, cannabidiolic acid; CINV, chemotherapy-induced nausea and vomiting; COX, cyclooxygenase; D_2_, dopamine 2; FAAH, fatty acid amide hydrolase; IC, insular cortex; i.p., intraperitoneal; LiCl, lithium chloride; NK1, neurokinin 1; MAGL, monoacylglycerol lipase; OEA, oleoylethanolamide; PEA, palmitoylethanolamine; PPARα, peroxisome proliferator-activated receptor alpha; s.c., subcutaneous; THCA, tetrahydrocannabinolic acid; TRPV1, transient receptor potential cation channel subfamily V member 1*.

### Acute nausea-induced conditioned gaping

Although rats cannot vomit, they display conditioned gaping reactions to a taste previously paired with an illness-inducing agent such as LiCl (Grill and Norgren, 1978). Only emetic drugs produce, and anti-emetic treatments (including cannabinoids) block conditioned gaping (see Parker, 2014 for review). Therefore, acute nausea-induced conditioned gaping is a reliable model of acute nausea in rats.

### Contextually elicited conditioned gaping, a preclinical model of anticipatory nausea

Rats also display conditioned gaping upon re-exposure to a nausea-paired context; this model is similar to the development of anticipatory nausea in humans (Limebeer et al., 2008). Furthermore, much like with human anticipatory nausea, a 5-HT_3_ receptor antagonist does not reduce contextually elicited conditioned gaping (Limebeer et al., 2006; Rock et al., 2014). Humans are treated with nonspecific benzodiazepines for anticipatory nausea, similarly, benzodiazepines reduce contextually elicited conditioned gaping in rats (Rock et al., 2014). Therefore, there is face validity for contextually elicited gaping as a preclinical model of anticipatory nausea.

### The role of the interoceptive insular cortex in conditioned gaping

Because the specific brain region(s) critical for nausea are still unclear, we are investigating the role of the endogenous cannabinoid system in nausea using the conditioned gaping model. One region of interest is the interoceptive insular cortex (IC), an area involved in the sensation of nausea in humans (Penfield and Faulk, 1955), as stimulation of the interoceptive IC (Ostrowsky et al., 2000; Isnard et al., 2004; Catenoix et al., 2008) and functional neuroimaging studies in humans (Napadow et al., 2013; Sclocco et al., 2014), pinpoint the interoceptive IC as a region critical for nausea.

#### Effect of Δ^9^-THC, THCA, and Δ^9^-THC-like synthetics on nausea

##### Acute nausea

Δ^9^-THC (0.5, 1, 10 mg/kg, i.p.), HU210 (0.001, 0.005 mg/kg, i.p.), and THCA (0.05, 0.5 mg/kg, i.p.) reduced acute nausea-induced conditioned gaping; an effect blocked by SR141716 (2.5 mg/kg, i.p.) (Parker and Mechoulam, 2003; Rock et al., 2013, 2015a).

##### Anticipatory nausea

Δ^9^-THC (0.5 mg/kg, i.p.) also reduced contextually elicited conditioned gaping (Limebeer et al., 2006; Rock et al., 2014), as did THCA (0.05 mg/kg, i.p), these effects were blocked by SR141716 (2.5 mg/kg, i.p.; Rock et al., 2013). These results suggest that CB_1_ receptor agonism reduces acute and anticipatory nausea in rats. However, the potential of CB_2_ receptor, TRPV1 receptor and PPARα antagonism to reduce the anti-nausea effects of THC or THCA have not been evaluated.

#### Effect of CBD and CBDA on nausea

##### Acute nausea

CBD (5 mg/kg, i.p. or s.c.) or CBDA (0.5 μg/kg–0.1 mg/kg, i.p.) reduced acute nausea-induced conditioned gaping (Parker and Mechoulam, 2003; Rock et al., 2012), these effects were blocked by a 5-HT_1A_ receptor antagonist (Rock et al., 2012, 2015a; Bolognini et al., 2013; Rock and Parker, 2013a). When combined with a low dose of ondansetron (1 μg/kg, i.p.), a subthreshold dose of CBDA (0.1 μg/kg, i.p.) enhanced the suppression of nausea-induced conditioned gaping (Rock and Parker, 2013a).

##### Anticipatory nausea

CBD (1, 5 mg/kg, i.p.) or CBDA (0.001, 0.01, 0.1 mg/kg, i.p.) suppressed contextually elicited gaping in the absence of any locomotor impairments (Rock et al., 2008, 2014; Bolognini et al., 2013), these effects were all reversed by a 5-HT_1A_ receptor antagonist (Bolognini et al., 2013). These results suggest a 5-HT_1A_ receptor mediated effect for CBD and CBDA in acute and anticipatory nausea and also a synergistic potential when combined with other anti-emetic agents.

#### Effect of AEA and FAAH inhibition on nausea

##### Acute nausea

FAAH inhibition (by PF3845, but not URB597) reduces acute nausea by a PPARα mechanism of action, not a CB_1_ receptor mechanism (Rock et al., 2015b). Previous work suggested that URB597 in combination with AEA also reduced LiCl-induced aversive responding, but not gaping *per se* (Cross-Mellor et al., 2007). The potential of TRPV1 or CB_2_ receptor antagonists to reverse the anti-nausea effects of FAAH inhibition has not yet been evaluated. It is interesting that elevated OEA and PEA occur in serum of patients when they are experiencing cyclical vomiting (Venkatesan et al., 2016), suggesting that they may be playing a homeostatic protective role. Current investigations are underway to determine if the anti-nausea effects of FAAH inhibition (possibly by a PPARα mechanism of action) are peripherally or centrally mediated.

##### Anticipatory nausea

In the preclinical model of anticipatory nausea, both URB597 (0.3, 10 but not 0.1 mg/kg, i.p.) and PF3845 (10 and 20 mg/kg, i.p.) suppressed the expression of previously established contextually elicited gaping, with both effects blocked by CB_1_ receptor antagonism, but not PPARα antagonism (Rock et al., 2008, 2015b). In addition, the selective FAAH inhibitor, AM4303 (20 mg/kg, i.p.), also reduced contextually-elicited conditioned gaping, with an increase in interoceptive IC AEA levels (Parker et al., 2016). These results suggest that FAAH inhibition may reduce anticipatory nausea through a CB_1_ receptor mediated effect; however, the potential of TRPV1 receptor antagonists and CB_2_ receptor agonists to reverse LiCl-induced anticipatory nausea expression has not yet been evaluated.

#### Effect of 2-AG and MAGL inhibition on nausea

##### Acute nausea

Exogenous 2-AG (1.25, 2 mg/kg, i.p.) suppressed acute nausea-induced conditioned gaping; this effect was blocked by cyclooxygenase (COX) inhibition (but not CB_1_ or CB_2_ antagonism; Sticht et al., 2012). When combined with the MAGL inhibitor JZL184 (40 mg/kg, i.p.), 2-AG (2 mg/kg, i.p.) suppressed acute nausea. Since this effect was reversed by AM251 (Sticht et al., 2012), prolonging 2-AG's duration of action (by MAGL inhibition) prevents the nausea produced by longer acting LiCl by acting at the CB_1_ receptor. In addition, the MAGL inhibitors MJN110 (10, 20 mg/kg, i.p.) or AM4301 (20 mg/kg, i.p.) reduced acute nausea-induced conditioned gaping, both effects were blocked by SR141716 (1 or 2.5 mg/kg, i.p.; Parker et al., 2015, 2016).

Intracranial administration of MAGL inhibitors (MJN110 [2 μg] or AM4301 [2 μg]), but not FAAH inhibitors (URB597 [0.01 μg] or PF3845 [2 μg]) into the interoceptive IC reduced acute nausea-induced conditioned gaping (Parker et al., 2016; Sticht et al., 2016) by a CB_1_ receptor mechansim of action (Sticht et al., 2016). Furthermore, selective increases in interoceptive IC 2-AG levels were detected following systemic (20 mg/kg, i.p.) or intra-interoceptive IC infusions of MJN110 (2 μg; Sticht et al., 2016). Interestingly, MJN110 (10 mg/kg, i.p.) reduced LiCl-induced increased c-Fos immunoreactivity in the interoceptive IC (Sticht et al., 2016). Finally, systemic injection of LiCl selectively elevated 2-AG levels, but not AEA, in the interoceptive IC. These data suggest that 2-AG acts as an endogenous anti-nausea compound in the interoceptive IC.

##### Anticipatory nausea

MJN110 (10, 20 mg/kg, i.p.) also reduced contextually-elicited conditioned gaping (with elevated interoceptive IC 2-AG levels), an effect blocked by SR141716 (1 mg/kg, i.p.; Parker et al., 2015). Furthermore, intra-interoceptive IC, MJN110 (2 μg, but not PF3845, nor ondansetron) suppressed contextually elicited conditioned gaping, blocked by CB_1_ receptor antagonism (Limebeer et al., 2016). The MAGL inhibitor, AM4301 (10, 20 mg/kg, i.p.), also reduced contextually elicited conditioned gaping, with a selective increase in interoceptive IC 2-AG levels (Parker et al., 2016). These results suggest 2-AG (but not AEA) reduces anticipatory nausea in the interoceptive IC, as well as acute nausea.

#### Effect of dual FAAH/MAGL inhibition on nausea

##### Acute nausea

The dual FAAH/MAGL inhibitor AM4302 (20 mg/kg, i.p.) suppressed acute nausea-induced conditioned gaping (Parker et al., 2016). Intra-interoceptive IC administration of the dual inhibitor JZL195 (10 μg) also suppressed acute nausea-induced conditioned gaping (Sticht et al., 2016).

##### Anticipatory nausea

Systemic administration of JZL195 (10 mg/kg, i.p.) also suppressed contextually elicited gaping, an effect blocked by SR141716 (2.5 mg/kg, i.p.; Limebeer et al., 2014). The dual inhibitor AM4302 (5, 10, 20 mg/kg, i.p.) was more effective than a FAAH (AM4303) or MAGL inhibitor (AM4301) in reducing contextually elicited gaping, an effect blocked by SR141716 (2.5 mg/kg, i.p), with a concomitant increase in 2-AG and AEA in the interoceptive IC (Parker et al., 2016). Therefore, dual FAAH/MAGL inhibition may boost the anti-nausea effects of elevation of 2-AG or AEA on their own for the treatment of anticipatory nausea.

## Conclusions

Animal models suggest that, in general, Δ^9^-THC, THCA, CBD, and CBDA, and manipulations of the endogenous cannabinoid system, have anti-emetic and anti-nausea properties. However, 2-AG and AEA's role in emesis is inconsistent across species. Further investigation is needed regarding the potential role of TRPV1 receptors in the anti-nausea effects produced by treatments that elevate AEA. It is time to take some of the preclinical findings (in particular CBDA, FAAH, and MAGL inhibition) into clinical trials for the treatment of acute and anticipatory nausea.

## Author contributions

ER wrote the article; LP edited the article.

## Funding

This research was funded by research grants from the Natural Sciences and Engineering Research Council of Canada (92057) and Canadian Institutes of Health Research (137122) to LP and Dr. Keith Sharkey.

### Conflict of interest statement

The authors declare that the research was conducted in the absence of any commercial or financial relationships that could be construed as a potential conflict of interest.
